# Anxiety and Depression-Related Factors in Hospitalized Patients Diagnosed With Coronavirus Disease 2019: A Detailed Cross-Sectional Analysis From a Tertiary Center

**DOI:** 10.7759/cureus.77865

**Published:** 2025-01-23

**Authors:** Arzu Kaplanoglu, Arzu Sonmez, Oznur Yildirim, Feride Cogullular, Elif Yurtsever, Birsen Oz, Hanife D Karabulut, Omer Akburak, Akif Erbin, Savas Ozturk

**Affiliations:** 1 Department of Nursing Management, Istanbul University – Cerrahpasa, Cerrahpasa Faculty of Medicine, Institute of Graduate Studies, Istanbul, TUR; 2 Department of Child Development, Beykent University, Faculty of Health Sciences, Istanbul, TUR; 3 Department of Nursing, University of Health Sciences Turkey, Haseki Training and Research Hospital, Istanbul, TUR; 4 Department of Urology, University of Health Sciences Turkey, Haseki Training and Research Hospital, Istanbul, TUR; 5 Division of Nephrology, Istanbul Faculty of Medicine, Istanbul, TUR

**Keywords:** anxiety, covid-19, depression, mental health, pandemic

## Abstract

Aim

During pandemic periods, psychogenic assessment and precautions are critical for patients requiring hospitalization. This study investigates the factors influencing anxiety levels in patients hospitalized for coronavirus disease 2019 (COVID-19) and analyzes the impact of demographic, social, and medical variables on anxiety and depression levels.

Methods

The research involved 150 female and 180 male patients hospitalized and treated in the adult pandemic service of a tertiary referral center in Istanbul from November 5, 2020, to February 5, 2021. Data were collected from patients on the third day of hospitalization by face-to-face or room-phone interviews. The study employed a hospital anxiety and depression scale together with sociodemographic information forms.

Results

Statistically significant positive associations were observed between anxiety and depression scores and age, as well as the severity of shortness of breath. Conversely, negative associations were identified with educational status. Female patients exhibited significantly higher scores in both categories compared to men. Illiterate individuals exhibited significantly higher anxiety scores than those who graduated from middle and high school. Non-working individuals exhibited significantly higher anxiety and depression scores compared to their working counterparts. Patients with chronic kidney disease, malignancy, and chest pain exhibited significantly elevated anxiety and depression scores relative to those without these conditions. Regarding room type, anxiety and depression scores were significantly higher in single rooms compared to double rooms and in the presence of a companion compared to being alone. In the logistic regression model, the primary anxiety risk factors identified include non-working status, a negative perception of the effectiveness of protective equipment, occupancy of single rooms, and fear of death. The main risk factors for depression identified were fear of death, a heightened risk among individuals with insufficient knowledge compared to those with adequate knowledge, absence of companionship, and a lack of educational information.

Conclusions

In patients who were illiterate, unemployed, lacked sufficient knowledge, held negative perceptions about protective equipment, experienced fear of death, resided in a single room, and were without an accompanying individual, both infection management and mental health must be addressed with sensitivity, and programs for psychological support should be formulated. The study's findings could potentially guide future pandemics.

## Introduction

Coronavirus disease 2019 (COVID-19) was first identified in Wuhan, the capital of China's Hubei region, in November 2019, and has spread rapidly to different parts of the world [1]. In March 2020, the disease was declared a pandemic by the World Health Organization [2]. This virus, which mainly causes respiratory tract infections, not only threatens the physical health of individuals but can also have both short-term and long-term effects on mental health [3]. Although being sick, hospitalized, and being a patient's relative during the pandemic phase are all similar experiences, the inability to welcome companions or visits to clinics and intensive care units distinguishes this process from a psychogenic one. At the same time, elements such as the spread of unpleasant and unhappy news about the pandemic process, particularly through the media and other communication means, as well as the intensive bustle of the pandemic agenda, enhance personal and mass stress, resulting in acute panic.

COVID-19 is characterized not only as a medical health crisis but also as a mental health emergency [4]. The unexpectedly wide spread of the disease, as well as an increasing number of cases and deaths, can cause psychological issues such as anxiety, depression, and stress in both health workers and the general public [5]. Many factors, such as personality traits, socioeconomic conditions, previous chronic mental or physical illness, the presence of social support, the ability to cope with the crisis and adapt to the new situation, and the psychological resilience of individuals, affect the reactive response to the process. At the same time, the way people interpret the images and news about the epidemic in the media, their confidence, insecurity, hopelessness, and thoughts about the future and the world can also be determinants of their response to the crisis they are experiencing [6]. For this reason, to manage this process effectively, it is important to know the psychosocial problems and underlying factors experienced by patients and to develop new process-specific approaches [7]. Understanding the psychological dimensions of the illness and implementing preventative measures can facilitate rehabilitation for both the patient and their companion.

Those with severe diseases or who are deemed to be risky patients are hospitalized for treatment. Psychosocial problems are expected to be more pronounced in these COVID-19 patients. Therefore, the psychogenic evaluation of hospitalized patients is critical. In the present study, it was aimed to examine the factors associated with the anxiety levels of patients hospitalized with the diagnosis of COVID-19 and to evaluate how demographic, social, and medical variables, such as age, gender, educational status, having a job, and having a chronic disease, affect anxiety.

## Materials and methods

Compliance with ethical standards

For the study, research permission from the Republic of Turkey Ministry of Health, ethics committee approval (Health Sciences University Turkey, Haseki Training and Research Hospital, Clinical Research Ethics Committee; date: 04.11.2020, decision no. 206), and institutional review board approval were obtained from the hospital where the research was conducted (Health Sciences University, Haseki Training and Research Hospital). The verbal and written informed consent of all participants was obtained.

Study design

The study included 150 female and 180 male patients who were hospitalized and treated in the adult pandemic service between 5.11.2020 and 5.02.2021 with the diagnosis of COVID-19 at the Istanbul Health Sciences University, Haseki Training and Research Hospital. Of these, patients hospitalized in the intensive care unit and patients who could not be contacted due to severe illness or severe neurological conditions were excluded from the study. The study data were collected from patients on the third day of hospitalization through in-person or room-phone interviews. A hospital anxiety and depression scale and a sociodemographic information form were used in the study.

The Hospital Anxiety and Depression Scale

The Hospital Anxiety and Depression Scale (HADS) was developed by Zigmond and Snaith in 1983 to determine the risk of anxiety and depression in patients and to measure their level and change in severity [8]. A validity and reliability study of the scale in Turkey was carried out by Aydemir et al. in 1997 [9]. This scale is widely used to determine the anxiety-depression risk group for those who apply to health institutions. Seven of the 14 questions (odd numbers) measure anxiety, and seven (even numbers) measure depression. Responses are scored between 0 and 3 on a Likert scale. The scoring of each item on the scale is different. Items 1, 3, 5, 6, 8, 10, 11, and 13 show decreasing severity, and the scoring is 3, 2, 1, 0. On the other hand, items 2, 4, 7, 9, 12, and 14 are scored as 0, 1, 2, and 3. For the anxiety subscale, items 1, 3, 5, 7, 9, 11, and 13 were collected; for the depression subscale, the scores of items 2, 4, 6, 8, 10, 12, and 14 were added. The lowest score that patients can get from both subscales is 0, and the highest score is 21. The cut-off points of the Turkish version of the HADS were calculated as 10 for the anxiety subscale (HADS-A) and 7 for the depression subscale (HADS-D).

Sociodemographic Data Form

This form, which was prepared by the researchers, includes a total of 25 questions, based mainly on age, gender, educational status, presence of chronic disease, psychiatric disease status, presence of psychiatric medication used, COVID-19 symptoms, severity, and type of room in the hospital.

Statistical analysis

IBM SPSS Statistics for Windows, Version 26.0 (released in 2019 by IBM Corp., Armonk, NY: IBM Corp.) was used for statistical analysis. Numbers and percentages are given for categorical variables; mean, standard deviation, minimum, maximum, median, and range are given for numerical variables. Comparisons of numerical variables in independent groups were made with the Mann-Whitney U test when the normal distribution condition was not met and with the Kruskal-Wallis test in groups of more than two. Subgroup analyses were performed with the Mann-Whitney U test and interpreted with Bonferroni correction. The rates in the groups were compared with the chi-square test. Risk factors associated with the development of anxiety were analyzed by logistic regression analysis. The statistical alpha significance level was accepted as p < 0.05.

## Results

Demographic and clinical data and hospitalization characteristics

The study included 330 patients, comprising 150 women and 180 men. The mean age was 55.7±15.7 years. Table 1 presents the patient's demographic characteristics, comorbidities, and hospitalization information.

**Table 1:** Demographic characteristics, comorbidities, and hospitalization information of the patients

Age, mean ± sd	55.7±15.7
Sex, n, %: Female	150 (45.5%)
Male	180 (54.5%)
Marital Status, n, %; Single	47 (14.2%)
Married	283 (85.8%)
Having Kids, n, %: Yes	280 (84.8%)
No	50 (15.2%)
Education Status, n, %: Illiterate	59 (17.9%)
Primary school	139 (42.1%)
Middle-High school	90 (27.3%)
University and above	42 (12.7%)
Employment Status, n, %: Employed	146 (44.2%)
Unemployed	184 (55.8%)
Income, n, %: Income less than expenses	107(32.4%)
Income equals expenses	190 (57.6%)
Income greater than expenses	33 (10%)
Comorbidity, n, %: No	138 (41.8%)

Analysis of the study group’s characteristics related to COVID-19 revealed that 98.8% needed hospital care due to symptoms. The predominant complaints included shortness of breath at 34.4%, cough at 24.2%, and fever at 23%. Additionally, 36.7% reported mild dyspnea while 30.9% described their dyspnea as moderate (Table 2). In 60.9% of the patients, there was the presence of shortness of breath along with respiratory distress, or SpO2 below 93% in room air, and a respiratory rate of 24 breaths per minute. Of the patients, 79.7% have received corticosteroids, 88.8% have demonstrated infiltration in lung computed tomography (CT) scans, and 77.6% have been diagnosed with bilateral infiltrations. The mean C-reactive protein (CRP) level was 56 mg/L (range: 22.8-120) while the average ferritin level was 330 ng/dL.

**Table 2:** Characteristics of patients related to COVID-19 disease * Mild: Definition of occasional shortness of breath, Moderate: Definition of shortness of breath on movement, Severe: Definition of shortness of breath in sitting and lying position

		n	%
Complaint	None	4	1.2
	Present	326	98.8
Types of Complaints	Fever	76	23
	Cough	80	24.2
	Sore throat	2	0.6
	Chest pain	9	2.7
	Shortness of breath	114	34.5
	Fatigue	71	21.5
	Myalgia-Joint pain	36	10.9
	Loss of appetite-Nausea	33	10
	Headache	34	10.3
	Loss of taste and smell	8	2.4
	Diarrhea	5	1.5
Shortness of Breath*	No	63	19.1
	Mild	121	36.7
	Moderate	102	30.9
	Severe	44	13.3
Hospital Room Type	Single room	117	35.5
	Double room	213	64.5
Hospital Companion	Yes	172	52.1
	No	158	47.9
Fear of Contagion	Yes	276	83.6
	No	54	16.4
Protective Equipment Effect	No effect	130	39.4
	Positive	186	56.4
	Negative	14	4.2
Fear of Death	Yes	169	51.2
	No	161	48.8
Knowledge Status	Had no knowledge	272	82.4
	Had sufficient knowledge	58	17.6
Hospitalization Time	1-5 days	77	23.3
	6-10 days	222	67.3
	16 days and above	31	9.4

Data-related measurements

In the study group, 83.3% reported receiving training in the hospital while 98.2% indicated satisfaction with the services offered. The mean anxiety score among the participating patients was 7.49±4.39 while the mean depression score was 8.96±4.56. Analysis revealed that 24.8% of the participating patients exhibited an anxiety score of 10 or higher while 52.7% demonstrated a depression score of 8 or higher. The cases involved exhibited elevated scores for anxiety and depression, suggesting an increased risk.

A strong positive correlation exists between anxiety scores and depression scores (r = 0.749, p < 0.001). Statistically significant positive associations were observed between anxiety and depression scores and age, as well as the severity of shortness of breath. Conversely, negative associations were identified with educational status (Table 3).

**Table 3:** Correlation analyses of anxiety and depression and study parameters CRP: C-reactive protein

	Anxiety		Depression	
	r	p	r	p
Depression	0.749	<0.001		
Age	0.124	0.024	0.228	<0.001
Education status	-0.157	0.004	-0.265	<0,001
Complaint of shortness of breath	0.211	<0.001	0.156	0.004
CRP mg/L	-0.015	0.780	0.079	0.150
Ferritin ng/ml	-0.078	0.161	0.031	0.575
Hospitalization time	-0.035	0.524	0.023	0.684

Analysis of the relationship between anxiety and depression scores and various study parameters revealed that women exhibited significantly higher scores in both categories compared to men. Differences in anxiety and depression scores were statistically significant across varying educational levels. Illiterate individuals exhibited significantly higher anxiety scores than those who graduated from middle and high school. Unemployed individuals exhibited significantly higher anxiety and depression scores compared to their working counterparts (p < 0.001). Individuals with chronic diseases exhibited significantly lower depression scores than those without chronic illnesses (p = 0.003). Participants with chronic kidney disease, malignancy, and chest pain exhibited significantly elevated anxiety and depression scores relative to those without these conditions. Significant differences in anxiety and depression scores were observed among different severity groups of shortness of breath (p < 0.001) (Table 4).

**Table 4:** Relationship between anxiety and depression scores and study parameters *Severe: Shortness of breath with respiratory distress or SPO2 below 93% in room air and respiratory rate of 24 or more per minute **Mild: Definition of occasional shortness of breath, Moderate: Definition of shortness of breath while moving, Severe: Definition of shortness of breath in sitting and lying position) CT: computed tomography

		Anxiety						Depression					
		Ort.	SD	Median	IQR		p	Ort.	SD	Median	IQR		p
Gender	Female	8.50	4.36	9	5	12	<0.001	9.79	4.53	9	6	13	0.007
	Male	6.65	4.24	6	3	9		8.28	4.48	8	5	11	
Marital status	Single	7.09	4.45	6	3	10	0.424	7.89	3.86	8	5	10	0.070
	Married	7.56	4.38	7	4	11		9.14	4.65	9	6	13	
Having kids	Yes	7.45	4.44	7	4	10.75	0.677	9.6	4.66	9	6	13	0.372
	No	7.72	4.13	7	5	10.25		8.40	3.99	8	6	11	
Education status	Illiterate	9.12	4.42	9	7	12	0.008	11.15	4.82	12	8	14	<0.001
	Primary school	7.47	4.58	7	4	10		9.17	4.63	9	6	12	
	Middle-High school	6.60	3.87	6	3	10		8.11	3.91	8	5	10	
	University and above	7.19	4.28	7	4	10		7.02	4.04	6	4	9.25	
Employment status	Employed	6.55	4.05	6	3	9	0.001	7.84	4.25	7	5	11	<0.001
	Unemployed	8.24	4.51	8	4.25	11		9.85	4.61	10	6	13	
Income	Income less than expenses	7.59	4.61	8	4	11	0.890	9.25	4.82	10	5	13	0.323
	Income equals expenses	7.51	4.38	7	4	10		8.97	4.40	9	6	12	
	Income greater than expenses	7.09	3.78	7	4	9		8	4.62	8	5	11	
Presence of chronic disease	No	7.02	4.42	7	3	10	0.112	8.09	4.73	7.5	4	12	0.003
	Yes	7.83	4.35	8	4	11		9.59	4.34	9	6	13	
Hypertension	No	7.29	4.45	7	4	10	0.212	8.80	4.62	9	5	12	0.298
	Yes	7.89	4.26	8	5	11		9.28	4.44	9	6	13	
Diabetes mellitus	No	7.40	4.41	7	4	10.5	0.494	8.9	4.67	9	5	13	0.575
	Yes	7.76	4.34	8	4	10.5		9.15	4.25	9	6.5	11.5	
Chronic obstructive pulmonary disease	No	7.39	4.43	7	4	10	0.135	8.88	4.57	9	6	12	0.270
	Yes	8.62	3.72	8.5	6.50	11.25		9.96	4.44	10	7.5	12	
Heart disease	No	7.54	4.38	7	4	10.75	0.318	9.02	4.53	9	6	12	0.349
	Yes	6.61	4.68	5	2.75	9.5		8	5.09	8	4.75	10.25	
Chronic kidney failure	No	7.33	4.35	7	4	10	0.002	8.84	4.58	9	5	12	0.017
	Yes	10.87	3.87	12	9	12		11.47	3.40	10	8	15	
Malignancy	No	7.43	4.36	7	4	10	0.018	8.91	4.55	9	6	12	0.028
	Yes	14.00	3	14	11	17		14.33	1.15	15	13	15	
Other	No	7.47	4.38				0.832	8.94	4.59				0.72
	Yes	7.84	4.69					9.32	4.22				
Status of hospitalization	Yes	7.66	4.26	8	4	10.5	0.249	8.98	4.53	9	6	12	0.847
	No	7..8	4.62	7	3	10.5		8.94	4.64	9	5	12.5	
Psychiatric disease	No	7.37	4.32	7	4	10	0.235	8.96	4.54	9	6	12	0.937
	Yes	8.5	4.84	8	5	12		9	4.84	9	5	12.75	
Psychiatric drug	Yes	8.83	4.89				0.233	9.13	4.28				0.988
	No	7.39	4.34					8.95	4.59				
Complaint	No	5.75	8.02	3	0	14.25	0.306	5.25	6.85	3	0.25	12.5	0.137
	Yes	7.51	4.34	7	4	10.25		9.01	4.52	9	6	12	
Fever	No	7.62	4.29	8	4	11	0.217	9.13	4.54	9	6	12	0.229
	Yes	7.07	4.70	6	3.25	10		8.42	4.63	7.50	4.25	13	
Cough	No	7.48	4.36	7	4	10.25	0.997	8.84	4.64	9	5	12	0.360
	Yes	7.53	4.51	7.5	4	10.75		9.36	4.31	9	6	12	
Sore throat	No	7.5	4.39	7	4	10.75	0.806	8.96	4.58	9	6	12	0.735
	Yes	6.50	4.95	6.5	3	10		9.5	0.71	9.5	9	10	
Chest pain	No	7.41	4.41	7	4	10	0.032	8.88	4.56	9	6	12	0.044
	Yes	10.22	2.54	10	8.5	12.5		11.78	4.06	14	8.5	15	
Shortness of breath	No	7.28	4.41	7	4	10.75	0.195	8.92	4.66	9	5	12	0.847
	Yes	7.89	4.34	8	4	10.25		9.04	4.4	9	6	12	
Fatigue	No	7.63	4.51	7	4	11	0.253	8.93	4.68	9	6	13	0.844
	Yes	7	3.92	7	4	9		9.10	4.15	9	6	12	
Myalgia-joint pain	No	7.41	4.4	7	4	10	0.305	8.94	4.48	9	6	12	0.693
	Yes	8.14	4.28	8.5	5.25	11		9.19	5.28	10	5	13	
Loss of appetite-nausea	No	7.46	4.44	7	4	11	0.669	8.89	4.6	9	6	12	0.241
	Yes	7.73	3.96	7	5	10		9.67	4.2.	10	6	13	
Headache	No	7.57	4.46	7	4	11	0.405	8.96	4.61	9	6	12	0.974
	Yes	6.82	3.76	7	4	9		8.97	4.18	8.5	6	12	
Loss of taste and odor	No	7.44	4.32	7	4	10	0.418	8.93	4.45	9	6	12	0.864
	Yes	9.63	6.55	8	4	16.5		10.13	8.37	6	3.5	19.75	
Diarrhea	No	7.5.	4.4	7	4	10.5	0.691	8.97	4.57	9	6	12	0.853
	Yes	6.6	4.34	6	3	11		8.6	4.22	8	5	12.5	
Shortness of breath severity**	No	5.46	4.12	5	2	8	<0.001	6.97	4.14	7	3	10	0.001
	Mild	7.48	4.22	7	4	11		9.35	4.71	9	6	13	
	Moderate	8.48	4.14	9	6	11		9.81	3.82	10	7	12.25	
	Severe	8.14	4.92	8	4	12		8.8	5.51	8.5	4	13.75	
Room type	Single room	8.77	4.84	9	5	13	<0.001	10.26	4.87	10	6	14	<0.001
	Double room	6.79	3.96	7	4	9		8.25	4.23	8	5	11	
Status of hospital companion	Yes	8.39	4.38	9	5	11	<0.001	10.42	4.56	10	7	14	<0.001
	No	6.51	4.2	6	3	9		7.37	4.01	7	4	10	
Fear of contagion	Yes	7.85	4.35	8	4	11	<0.001	9.17	4.48	9	6	12	0.062
	No	5.67	4.19	5	2.75	8		7.89	4.87	7	4	12.25	
Effect of protective equipment	Had no effect	7.42	4.41	7	4	11	0.001	9.23	4.46	9	6	13	0.001
	Positive	7.20	4.26	7	3.75	10		8.45	4.53	8	5	11	
	Negative	11.93	3.65	11.5	9.75	14.25		13.29	3.60	13.5	11	15.25	
Fear of death	Yes	9.44	4.09	10	7	12	<0.001	11.07	4.24	11	8.5	14	<0.001
	No	5.45	3.72	5	2	8		6.75	3.79	6	4	9	
Knowledge status	Had no knowledge	7.62	4.5	7	4	11	0.288	9.38	4.60	9	6	13	<0.001
	Had sufficient knowledge	6.90	3.79	7	3.75	9		7.02	3.85	6.5	4	10	
Having education and information	Yes	8.08	4.34	8	5	11	<0.001	9.6	4.47	9	6	13	<0.001
	No	4.55	3.38	4	2	7		5.76	3.56	5	3	8	
Service satisfaction	Yes	7.51	4.38	7	4	10.75	0.516	8.99	4.57	9	6	12	0.406
	No												
Disease Severity*	Severe	7.82	4.48	8	4	11	<0.001	9.64	4.74	10	6	13	<0.001
	Not severe	6.98	4.21	7	4	10		7.91	4.07	8	5	11	
Taking corticosteroid	Yes	7.62	4.36	7	4	10	0.569	9.11	4.49	9	6	12	0.587
	No	7.28	4.6	7	3	11		8.78	4.93	8.5	5	13	
Presence of infiltration in CT	Yes	7.48	4.32	7	4	10	0.97	9.05	4.55	9	6	12	0.638
	No infiltration	7.57	5	7	3	11		8.30	4.67	9	3.5	13	
Is infiltration bilateral?	No infiltration	7.57	5	7	3	11	0.981	8.30	4.67	9	3.5	13	0.887
	Bilateral	7.47	4.32	7	4	10		9.06	4.70	9	6	12.75	
	Unilateral	7.54	4.31	7	4	11		8.97	3.37	9	6	11	

Regarding room type, anxiety and depression scores were significantly higher in single rooms compared to double rooms and in the presence of a companion compared to being alone. Anxiety and depression scores were significantly higher in participants who had a fear of death compared to those who did not and in participants who had a fear of transmission compared to those who did not. Anxiety scores were significantly higher in patients lacking educational information about the disease relative to those who received such information. Similarly, depression scores were significantly higher in participants who reported no knowledge of the disease compared to those who indicated sufficient knowledge (Table 4).

Significant differences in anxiety and depression scores were identified among groups with differing views on protective equipment. Individuals who perceived the protective equipment's effect negatively exhibited significantly higher anxiety and depression scores than those with a positive perception or those who believed it had no effect (Table 4).

Statistically significant differences were found in the rates of moderate to severe shortness of breath in individuals with Beck anxiety scores of 10 and above based on gender, unemployed status, chronic kidney disease (CKD), malignancy, and BECK anxiety score groups (p = 0.009). Individuals with Beck anxiety scores of 10 and above had higher rates of moderate to severe shortness of breath compared to those with scores below 10. Participants with Beck anxiety scores of 10 and above showed statistically significantly higher rates of being in single rooms, having a companion, having a fear of transmission, having a fear of death, lacking knowledge about the disease, and not receiving educational information compared to those with scores below 10 (fear of transmission p = 0.043, knowledge status p = 0.018, other comparisons p < 0.001). Significant differences were also observed in the perception of the effectiveness of protective equipment among the Beck anxiety score groups (p < 0.001). Participants with Beck anxiety scores of 10 and above had higher rates of believing that protective equipment had no effect (Table 5).

**Table 5:** Characteristics of patients with and without anxiety *Severe: Shortness of breath with respiratory distress or spo2 below 93% in room air and respiratory rate of 24 or more per minute **Mild: Definition of occasional shortness of breath, Moderate: Definition of shortness of breath while moving, Severe: Definition of shortness of breath in sitting and lying position) HT: hypertension; DM: diabetes mellitus; COPD: chronic obstructive pulmonary disease

		Beck anxiety				
		<10		10 and above		
		n	%	n	%	p
Gender	Female	88	39.3%	62	58.5%	0.001
	Male	136	60.7%	44	41.5%	
Marital Status	Single	32	14.3%	15	14.2%	0.974
	Married	192	85.7%	91	85.8%	
Having kids	Yes	191	85.3%	89	84.0%	0.757
	No	33	14.7%	17	16.0%	
Education status	Illiterate	33	14.7%	26	24.5%	0.095
	Primary school	93	41.5%	46	43.4%	
	Middle-High school	67	29.9%	23	21.7%	
	University and above	31	13.8%	11	10.4%	
Employment status	Employed	114	50.9%	32	30.2%	<0.001
	Unemployed	110	49.1%	74	69.8%	
Income	Income less than expenses	66	29.5%	41	38.7%	0.142
	Income equals expenses	132	58.9%	58	54.7%	
	Income greater than expenses	26	11.6%	7	6.6%	
Presence of chronic disease		130	58%	62	58.5%	0.938
	HT	72	32.1%	39	36.8%	0.404
	DM	59	26.3%	26	24.5%	0.725
	COPD-Asthma	17	7.6%	9	8.5%	0.777
	Heart disease	14	6.3%	4	3.8%	0.355
	Chronic renal failure	4	1.8%	11	10.4%	0.001
	Malignancy	0	0%	3	2.8%	0.033
	Other	14	6.3%	5	4.7%	0.577
Previous hospitalization		142	63.4%	71	67.0%	0.525
Psychiatric disease		24	10.7%	12	11.3%	0.869
Psychiatric drug		16	7.1%	7	6.6%	0.857
Complaint		221	98.7%	105	99.1%	1.000
	Fever	54	24.1%	22	20.8%	0.499
	Cough	56	25%	24	22.6%	0.641
	Sore throat	1	0.4%	1	0.9%	0.540
	Chest pain	4	1.8%	5	4.7%	0.153
	Shortness of breath	71	31.7%	43	40.6%	0.114
	Fatigue	58	25.9%	13	12.3%	0.005
	Myalgia, joint pain	24	10.7%	12	11.3%	0.869
	Loss of appetite nausea	23	10.3%	10	9.4%	0.814
	Headache	27	12.1%	7	6.6%	0.128
	Loss of taste/odor	5	2.2%	3	2.8%	0.715
	Diarrhea	3	1.3%	2	1.9%	0.658
Shortness of breath severity*	None	53	23.7%	10	9.4%	0.009
	Mild	83	37.1%	38	35.8%	
	Moderate	62	27.7%	40	37.7%	
	Severe	26	11.6%	18	17.0%	
Room type	Single room	63	28.1%	54	50.9%	<0.001
	Double room	161	71.9%	52	49.1%	
Presence of hospital companion	Yes	99	44.2%	73	68.9%	<0.001
	No	125	55.8%	33	31.1%	
Fear of contagion	Yes	181	80.8%	95	89.6%	0.043
	No	43	19.2%	11	10.4%	
Effect of protective equipment	Had no effect	84	37.5%	46	43.4%	<0.001
	Positive	137	61.2%	49	46.2%	
	Negative	3	1.3%	11	10.4%	
Fear of death		83	37.1%	86	81.1%	<0.001
Knowledge status	Had no knowledge	177	79%	95	89.6%	0.018
	Had sufficient knowledge	47	21%	11	10.4%	
Duration of hospitalization	1-5 days	48	21.4%	29	27.4%	0.169
	6-10 days	158	70.5%	64	60.4%	
	16 days and above	18	8,0%	13	12.3%	
Having education and information		174	77.7%	101	95.3%	<0.001
Service satisfaction		220	98.2%	104	98.1%	1.000
Disease severity**	Severe	129	57.6%	72	67.9%	0.072
	Not severe	95	42.4%	34	32.1%	
Taking corticosteroids status		176	81.5%	87	82.9%	0.764
Presence of infiltration in CT		199	88.8%	94	88.7%	0.966
Is infiltration bilateral?	Infiltrasyon, no	25	11.2%	12	11.3%	0.724
	Bilateral	176	78.6%	80	75.5%	
	Unilateral	23	10.3%	14	13.2%	

A model was developed to examine anxiety risk factors through univariate analyses, utilizing variables with p < 0.250. The primary factors identified include unemployed status, a negative perception of the effectiveness of protective equipment, occupancy of single rooms, and fear of death (Table 6).

**Table 6:** Multivariate logistic regression analysis of anxiety risk factors

	OR	%95 CI		p
Gender (Male/Female)	1.576	0.834	2.981	0.161
Education Status (Ref: Illiterate)				0.435
Primary School	1.458	0.657	3.234	0.354
Middle-High School	1.570	0.602	4.096	0.357
University and Above	2.966	0.809	10.866	0.101
Employment Status (Employed/Unemployed)	2.010	0.983	4.108	0.056
Income (Ref: Income less than expenses)				0.821
Income Equals Expenses	0.955	0.522	1.746	0.88
Income Greater Than Expenses	0.679	0.201	2.290	0.532
Chronic Kidney Failure	4.099	1.081	15.546	0.038
Room Type (Ref: Double vs Single room)	2.187	1.212	3.945	0.009
Hospital Companion (Ref: Yes) No	0.631	0.348	1.145	0.130
Fear of Contagion	1.115	0.468	2.658	0.806
Effect of Protective Equipment (Ref: had no effect)				0.034
Positive	0.649	0.365	1.154	0.141
Negative	3.705	0.859	15.979	0.079
Fear of Death	4.793	2.528	9.09	<0.001
Knowledge Status (Had sufficient knowledge/Had no knowledge)	1.905	0.681	5.333	0.220
Hospitalization Time (Ref: 1-5 days)				0.087
6-10 Days	0.513	0.261	1.009	0.053
16 Days and Above	1.008	0.362	2.812	0.987
Severity of the Disease (respiratory distress: no/yes)	0.957	0.518	1.767	0.887

A model was developed for the examination of depression risk factors through univariate analyses, utilizing variables with p < 0.250. The primary factors identified include fear of death, increased risk for individuals lacking knowledge compared to those with adequate knowledge, absence of companionship, and lack of educational information (Table 7).

**Table 7:** Depression risk factors multivariate logistic regression analysis * Mild: Definition of occasional shortness of breath, Moderate: Definition of shortness of breath on movement, Severe: Definition of shortness of breath in sitting and lying position)

	OR	%95 CI		p
Gender (Male/Female)	0.832	0.419	1.654	0.600
Education status (Ref: Illiterate)				0.840
Primary school	0.958	0.355	2.582	0.932
Middle-High school	0.811	0.262	2.511	0.717
University and above	0.591	0.152	2.302	0.448
Employment status (Employed/Unemployed)	1.240	0.604	2.549	0.558
Room type (Ref: Double vs Single room)	1.743	0.842	3.606	0.134
Hospital companion (Ref: Yes) No	0.656	0.348	1.238	0.193
Fear of contagion	0.594	0.262	1.348	0.213
Effect of protective equipment (Ref: Had no effect)				0.586
Positive	0.719	0.384	1.345	0.302
Negative			.	0.998
Fear of death	6.115	3.030	12.340	<0.001
Knowledge status (Had sufficient knowledge/Had no knowledge)	1.494	0.660	3.384	0.335
Hospitalization time (Ref:1-5 days)				0.480
6-10 days	0.770	0.365	1.625	0.493
16 days and above	1.486	0.403	5.476	0.551
Severity of the disease (Respiratory distress: no/yes)	0.887	0.458	1.716	0.721
Hypertension	0.617	0.303	1.256	0.183
Diabetes mellitus	1.859	0.845	4.090	0.123
Chronic obstructive pulmonary disease	0.873	0.257	2.973	0.828
Chronic kidney failure	2.251	0.237	21.384	0.480
Fever	0.860	0.415	1.782	0.685
Cough	1.644	0.794	3.405	0.181
Chest pain	2.308	0.166	32.086	0.533
Shortness of breath *				0.733
Mild	0.836	0.372	1.879	0.664
Moderate	1.104	0.430	2.834	0.837
Severe	0.644	0.216	1.921	0.430
Having education and information	0.166	0.070	0.392	<0.001
Status of taking corticosteroids	0.613	0.266	1.414	0.252

## Discussion

In our study, the anxiety and depression states of the patients hospitalized with the diagnosis of COVID-19 during the epidemic period were evaluated, and it was found that 24.7% of them were anxious and 52.7% had depression symptoms. In the first study reporting the prevalence of anxiety and depression in patients with COVID-19 in the city of Wuhan at the beginning of the epidemic, it was reported that 34.7% and 28.4% had symptoms of anxiety and depression, respectively [10]. Anxiety is affected by many factors such as environmental, emotional, and physical factors. For example, having to stay at home is one of the main factors that increases anxiety [11]. Although both studies are cross-sectional, the depression rate may have been low due to the fact that the study in Wuhan was conducted at the very beginning of the epidemic and that the extent of it could not be foreseen. We believe that the main factors that increase depression in our study are the prolongation of the epidemic and the feeling of hopelessness.

In our study, it was found that the rates of anxiety and depression increased with increasing age. The fact that the symptoms of COVID-19 are more severe and the risk of death is higher at older ages has been considered as the main reason for these increases [12]. When the relationship of anxiety symptoms with gender is examined, in parallel with the study of Metin et al., it is seen that it is more common in women [13]. Women who assume a protective role in the family and are more sensitive to separation are more prone to anxiety symptoms.

Consistent with the literature, in our study, anxiety symptoms were found to be higher as the level of education decreased [14]. On the contrary, some studies are showing that being educated and working increases the level of anxiety [15]. It is not surprising that education influences anxiety levels. Individuals with a high level of education can calm their psychological states more easily by accessing the right information [16]. In our study, anxiety and depression levels were also found to be higher in the unemployed population. It is thought that working in jobs that require or oppose protection measures, such as wearing masks, maintaining physical distance, and avoiding crowds, may cause different results.

Dyspnea is associated with interactions between multiple physiological, psychological, social, and environmental factors [17]. With the aggravation of respiratory distress, patients may consider shortness of breath a threat associated with anxiety or depressive symptoms. Therefore, dyspnea may be associated with anxiety or depressive symptoms during hospitalization in COVID-19 patients. However, the relationship between dyspnea and depressive symptoms has not been clearly demonstrated in the literature [18]. While individuals with chronic diseases are expected to have higher depressive symptoms than healthy individuals [19], it was observed that no higher depressive symptoms were found in individuals with chronic diseases.

Though social support is one of the critical factors associated with anxiety and depression for COVID-19 patients, the rates of anxiety and depression were found to be high in those with companions in our study. This situation can also be interpreted as asking for a companion because of high anxiety and depression. In addition, it was observed that being in a single room, accompanied by a fear of death, and using protective equipment increased the symptoms of anxiety and depression. Feelings of loneliness and helplessness and not being able to see their relatives due to isolation suggest that this may be the reason [20].

It was reported that only anxiety symptoms were higher in those who were fearful of contagion and those who received educational information, whereas depression findings were higher in those who had no knowledge [10]. In our study, in parallel with the literature, while anxiety was higher in those who received education and information, depression was higher in those who did not.

Our study had some limitations. The major limitations are that it was performed in a single center, and the possible effects of the drugs given in the treatment could not be evaluated. Despite these limitations, the study's strengths are its thorough examination of nearly all variables, including logistic regression analysis. Our study meticulously analyzed demographic, socioeconomic, and medical aspects. All factors potentially linked to depression and anxiety have been analyzed. The findings of our investigation will serve as a reference for potential future pandemic scenarios.

## Conclusions

Significant symptoms of anxiety and depression are observed during hospitalization in patients hospitalized due to COVID-19. In patients who were illiterate, unemployed, lacked sufficient knowledge, held negative perceptions about protective equipment, experienced fear of death, resided in a single room, and were without an accompanying individual, both infection management and mental health must be addressed with sensitivity, and programs for psychological support should be formulated. In addition, follow-ups and treatment schemes should also be organized for the psychological state of the discharged patients. The study's findings could potentially guide future pandemics.

## References

[1] Zhu N, Zhang D, Wang W (2020). A novel coronavirus from patients with pneumonia in China, 2019. N Engl J Med.

[2] (2020). World Health Organization. Coronavirus disease 2019 (COVID-19). Situation report. http://www.who.int/docs/default-source/coronaviruse/situation-reports/20200227-sitrep-38-covid-19.pdf?sfvrsn=9f98940c_2.on28February2020..

[3] Almond D, Mazumder B (2005). The 1918 influenza pandemic and subsequent health outcomes: an analysis of SIPP data. Am Econ Rev.

[4] Luo M, Guo L, Yu M, Jiang W, Wang H (2020). The psychological and mental impact of coronavirus disease 2019 (COVID-19) on medical staff and general public - a systematic review and meta-analysis. Psychiatry Res.

[5] Tönbül Ö (2020). Koronavirüs (Covid-19) Salgını Sonrası 20-60 Yaş Arası Bireylerin Psikolojik Dayanıklılıklarının Bazı Değişkenler Açısından İncelenmesi [Article in Turkish]. Humanistic Perspective.

[6] Jiang X, Deng L, Zhu Y (2020). Psychological crisis intervention during the outbreak period of new coronavirus pneumonia from experience in Shanghai. Psychiatry Res.

[7] Solmi M, Thompson T, Cortese S (2025). Collaborative outcomes study on health and functioning during infection times (COH-FIT): insights on modifiable and non-modifiable risk and protective factors for wellbeing and mental health during the COVID-19 pandemic from multivariable and network analyses. Eur Neuropsychopharmacol.

[8] Zigmond AS, Snaith RP (1983). The hospital anxiety and depression scale. Acta Psychiatr Scand.

[9] Kelleci M, Aydın D, Sabancıoğulları S, Doğan S (2009). Hastanede yatan hastaların bazı tanı gruplarına göre anksiyete ve depresyon düzeyleri [Article in Turkish]. Klinik Psikiyatri.

[10] Kong X, Zheng K, Tang M (2020). Prevalence and factors associated with depression and anxiety of hospitalized patients with COVID-19 [Pre-print]. MedRxiv.

[11] Abdelghani M, El-Gohary HM, Fouad E, Hassan MS (2020). Addressing the relationship between perceived fear of COVID-19 virus infection and emergence of burnout symptoms in a sample of Egyptian physicians during COVID-19 pandemic: a cross-sectional study. Middle East Curr Psychiatry.

[12] Yang X, Yu Y, Xu J (2020). Clinical course and outcomes of critically ill patients with SARS-CoV-2 pneumonia in Wuhan, China: a single-centered, retrospective, observational study. Lancet Respir Med.

[13] Metin A, Erbiçer ES, Şen S, Çetinkaya A (2022). Gender and COVID-19 related fear and anxiety: a meta-analysis. J Affect Disord.

[14] Megatsari H, Laksono AD, Ibad M, Herwanto YT, Sarweni KP, Geno RA, Nugraheni E (2020). The community psychosocial burden during the COVID-19 pandemic in Indonesia. Heliyon.

[15] Kiely KM, Brady B, Byles J (2019). Gender, mental health and ageing. Maturitas.

[16] Hurwitz B, Greenhalgh T, Skultans V (2008). Narrative Research in Health and Illness.

[17] Parshall MB, Schwartzstein RM, Adams L (2012). An official American Thoracic Society statement: update on the mechanisms, assessment, and management of dyspnea. Am J Respir Crit Care Med.

[18] Schuler M, Wittmann M, Faller H, Schultz K (2018). The interrelations among aspects of dyspnea and symptoms of depression in COPD patients - a network analysis. J Affect Disord.

[19] Gerontoukou EI, Michaelidoy S, Rekleiti M, Saridi M, Souliotis K (2015). Investigation of anxiety and depression in patients with chronic diseases. Health Psychol Res.

[20] Shaw SC (2020). Hopelessness, helplessness and resilience: the importance of safeguarding our trainees' mental wellbeing during the COVID-19 pandemic. Nurse Educ Pract.

